# Restoration of the prolyl-hydroxylase domain protein-3 oxygen-sensing mechanism is responsible for regulation of HIF2α expression and induction of sensitivity of myeloma cells to hypoxia-mediated apoptosis

**DOI:** 10.1371/journal.pone.0188438

**Published:** 2017-12-05

**Authors:** Gilberto Gastelum, Aleksandra Poteshkina, Mysore Veena, Edgar Artiga, Geraldine Weckstein, Patrick Frost

**Affiliations:** 1 Department of Hematology/Oncology, Greater Los Angeles Veteran Administration Healthcare System, Los Angeles, California, United States of America; 2 Department of Hematology/Oncology, University of California, Los Angeles, Los Angeles, California, United States of America; University of Catanzaro, ITALY

## Abstract

Multiple myeloma (MM) is an incurable disease of malignant plasma B-cells that infiltrate the bone marrow (BM), resulting in bone destruction, anemia, renal impairment and infections. Physiologically, the BM microenvironment is hypoxic and this promotes MM progression and contributes to resistance to chemotherapy. Since aberrant hypoxic responses may result in the selection of more aggressive tumor phenotypes, we hypothesized that targeting the hypoxia-inducible factor (HIF) pathways will be an effective anti-MM therapeutic strategy. We demonstrated that MM cells are resistant to hypoxia-mediated apoptosis *in vivo* and *in vitro*, and that constitutive expression of HIF2α contributed to this resistance. Since epigenetic silencing of the prolyl-hydroxylase-domain-3 (PHD3) enzyme responsible for the O_2_-dependent regulation of HIF2α is frequently observed in MM tumors, we asked if PHD3 plays a role in regulating sensitivity to hypoxia. We found that restoring PHD3 expression using a lentivirus vector or overcoming PHD3 epigenetic silencing using a demethyltransferase inhibitor, 5-Aza-2’-deoxycytidine (5-Aza-dC), rescued O_2_-dependent regulation of HIF2α and restored sensitivity of MM cells to hypoxia-mediated apoptosis. This provides a rationale for targeting the PHD3-mediated regulation of the adaptive cellular hypoxic response in MM and suggests that targeting the O_2_-sensing pathway, alone or in combination with other anti-myeloma chemotherapeutics, may have clinical efficacy.

## Introduction

The critical ability of tumor cells to overcome the physiologically limiting effects of low pO_2_ is primarily mediated by the hypoxia-inducible factors (HIFs) (for review see [1]). HIFs are composed of a constitutively expressed β-subunit (HIF1β/ARNT) and oxygen-responsive α-subunits (HIF1α, 2α, and 3α) whose expression is regulated by proteosome-mediated degradation. Under “normoxic” conditions (for most tissues pO_2_~85-150mmHg), the HIFα subunits are hydroxylated by a number of closely related prolyl-hydroxylase domain proteins (PHD1-3) that results in recognition of the α-subunit by the von Hippel-Lindau tumor suppressor (VHL), and their subsequent ubiquination and rapid degradation by the proteosome. However, HIF-mediated transcriptional activity is induced by hypoxic conditions (pO_2_~<50mmHg), during which the PHD activity is suppressed, and HIFα degradation is inhibited [2]. This “canonical” O_2_-dependent regulatory system can be bypassed or disrupted by gain of oncogene function or loss-of-function mutations in tumor-suppressor genes (i.e. phosphatase and tensin homolog (PTEN)), or epigenetic silencing of the O_2_-sensing pathways resulting in constitutive O_2_-independent stabilization and expression of the HIFα-subunits which can support tumor growth and survival [3–5].

The hypoxic microenvironment of the bone marrow has been implicated as playing a role in MM tumorogenesis [6] and chemoresistance [7], yet the exact underlying mechanisms by which this occurs remains unclear and poorly defined. We hypothesized that disruption of the O_2_-sensing pathway(s) and the constitutive activation of HIF may play a critical role in these events by inducing more malignant tumor phenotypes that facilitate migration and engraftment of tumor cells into the hypoxic marrow microenvironment and impact on MM pathophysiology. Highlighting this, we recently demonstrated that HIFα-subunits are differentially regulated by low pO_2_ in MM cell lines and that displacing HIF from its DNA-binding site [8] using a Pyrrole-Imidazole (Py-Im) polyamide compound (HIF-PA) inhibited a subset of HIF-activated genes and sensitized MM cells to hypoxia-mediated apoptosis *in vitro* and *in vivo* [9]. We also found that using a MM xenograft model, targeting HIF activity with polyamides decreased microvessel density and significantly increased the regions of necrosis and apoptosis within the tumor nodules. Finally, we also noted that the downregulation of the PHD3 isoform in these MM cell lines was correlated with the expression patterns of the HIF2α-subunit, but not the HIF1α subunit.

*PHD3* is a presumptive tumor suppressor gene that is distinct from the other PHD isoforms whose expression varies between different cell types and oxygen concentrations. PHD3 promotes neuronal apoptosis [10], and downregulation of PHD3 expression is frequently observed in glioblastoma [11, 12], colorectal cancer [13], soft tissue sarcomas [14] and breast cancer [15]. In some tumors, PHD3 expression is lost during the process of tumor de-differentiation and metastasis [13, 16]. The *PHD3* gene is silenced by CpG methylation of the PHD3 promoter in a subset of human carcinoma cell lines of diverse origin [17]. Finally, Shah et al [18] and Hatzimicheal et al [19] reported frequent aberrant CpG methylation of *PHD3* (but not *PHD2*) in MM patient samples (42% and 33% respectively) and this correlated to poor prognosis. While most of the studies of PHD3’s role in cancer have been performed using solid tumor models, it is unclear what role PHD3 deregulation plays in hematological malignancies engrafted in the hypoxic bone marrow. This raises important questions as to the roles played by the disruption of O_2_-sensing PHD regulatory pathways and the impact upon MM. Here we explore the regulatory role of PHD3 on HIF2α expression and its impact on hypoxia-mediated apoptosis in MM cells and demonstrate that restoring PHD3 expression using a lentivirus vector or treating MM cells with a demethyltransferase inhibitor rescues O_2_-dependent regulation of HIF2α in MM cells and that this is sufficient to sensitize MM cells to hypoxia-mediated apoptosis *in vitro*.

## Materials and methods

### Cell lines and reagents

All cell lines were purchased from ATCC and maintained at 37°C and 5% CO_2_ (“normoxic” condition) unless noted. The cell lines were validated using the Johns Hopkins Genetic Core Research Facility (Baltimore, MD) and stock aliquots were stored under liquid nitrogen. Testing for mycoplasma was performed using a mycoplasma PCR detection kit (Sigma-Aldrich, St Louis, MO). Cellular apoptosis was measured by flow cytometry using a cleaved caspase-3 kit or a annexin V/PI kit (BD Biosciences, San Jose, CA). Enzyme-linked immunosorbent assay (ELISA) kits specific for human VEGF was purchased from R&D Systems (Minneapolis, MN).

### Immunoblots

Protein was isolated and western blot analysis was performed as described previously [20]. Nuclear and cytoplasmic fractions were isolated using the Thermo Scientific NE-PER Nuclear and Cytoplasmic Extraction Kit (Rockford, IL) following the manufacturer’s instructions. HIF1α antibody (clone 54/HIF1) was purchased from BD Biosciences. β-tubulin (clone H-235), goat anti-mouse and goat anti-rabbit IgG horseradish peroxidase-conjugated antibodies were purchased from Santa Cruz Biotechnology (Dallas, TX). The PARP (clone 46D11) cleaved caspase 9 (clone Asp330), AKT-total (clone C67E7), AKT-S473 (clone D9E) and AKT-T308 (clone C31E5E), phospo-GSK3β (clone 27C10) antibodies and the P70-total, P70-T421/S424, and P70-T389 antibody kits were purchased from Cell Signaling Technology (Danvers, MA). The EGLN1/PHD2 (rabbit polyclonal), EGLN3/PHD3 (mouse polyclonal), EGLN3/PHD3 positive control (an EGLN3/PHD3 over expressing lysate from HEK293T cells), and HIF2α (rabbit polyclonal) were purchased from NOVUS Biologicals (Littleton, CO).

### Generation of PHD3 isogenic 8226 cell lines

The PHD3/EGLN3 open reading frame (ORF) (ref sequence NM_022073.3) was cloned into a lentivirus vector purchased from OriGene (Rockville MD). Isogenic 8226 cells were infected with the viral particles (empty vector controls or PHD3) at a multiplicity of infection (MOI) of 10 and gentamycin selection was started 72 hours later. Successful transduction was confirmed by immunoblot for PHD3 expression. Small inhibitory RNA (siRNA) for HIF1α (Silencer Select siRNA ID# s6539, gene ID# 3091), HIF2α (Silencer Select siRNA ID# s6539, gene ID# 2034), and scrambled control RNA (Silencer Select negative control #1 siRNA,) were purchased from Ambion (Grand Island, NY). Cells were transfected with siRNA using Lipofectamine-2000 (Life Technologies, Grand Island, NY).

### *5*-*Aza*-2'-deoxycytidine (5-Aza-dC) treatment

Cells were counted, and seeded (day 0) at 1 X 10^6^ cells/100 mm dish. Fresh 5-Aza-dC (Sigma-Aldritch, St Louis MO) (at concentration of 4 or 8 μM) was added to the dish on days 1, 3, and 5 while a control flask was treated only with vehicle control. On day 6, the cells were collected on ice, and RNA was harvested and analyzed as described below.

### Semi-quantitative RT-PCR (qRT-PCR)

Total RNA was extracted from individual cell lines using RNeasy Mini Kit (Qiagen, Valencia, CA) and quantified using a NanoDrop 1000. To assess *PHD3*, *PHD2*, *HIF1*α, *HIF2*α and GAPDH expression, 500 ng of total RNA was used for reverse transcription using a OneStep RT-PCR Kit (Qiagen). The *PHD3* forward primer is 5′-GGGCAAATACTACGTCAAGGAG-3′ and the reverse primer is 5′-AGTCTTCAGTGAGGGCAGATTC-3′. The *PHD2* forward primer is 5’-GGACGACCTGATACG-3’ and the reverse primer is 5’-ACTTACCTTGGCATCC. The *HIF1*α forward primer is 5’-CTCAAAGTCGGACAGCCTCA-3’ and the reverse primer is 5’-CCCTGCAGTAGGTTTCTGCT-3’. The *HIF2*α forward primer is 5’-GACATGAAGTTCACCTACTGTGATG-3’ and the reverse primer is 5’-GCGCATGGTAGAATTCATAGG-3’. *GAPDH* expression was assessed using GAPDH-specific primers. PCR conditions for *PHD3*, *PHD2*, *HIF1*α, *HIF2*α and *GAPDH* were the same except that 28 cycles of PCR were performed for *PHD3*, *PHD2*, *HIF1*α, *HIF2*α analysis and 23 cycles were performed for *GAPDH*. The parameters used were: 95°C for 5 minutes followed by the stated number of cycles of 94°C for 1 minute; 56°C for 1 minute, and 72°C for 1 minute, ending with a final extension at 72°C for 7 minutes. The amplified products were electrophoresed on a 1% agarose gel and stained with ethidium bromide to visualize the bands. Primers were purchased from Invitrogen (Carlsbad, CA).

### Statistics

Data was screened for consistency and quality by both graphical (histograms, scatter plots) and analytical methods (descriptive statistics). Variables were analyzed using generalized linear models (GLM) such as ANOVA and *t*-tests. The effect of culturing cells under hypoxic conditions in the presence of chemotherapy (bortezomib or rapamycin) on induction of apoptosis was assessed by the median effect method using Calcusyn Software Version 1.1 (Biosoft, Cambridge, United Kingdom). Combination indices (CI) values were calculated using the most conservative assumption of mutually nonexclusive treatment/drug interactions. CI values were calculated from median results of apoptosis assays.

## Results and discussion

**Fig 1:**
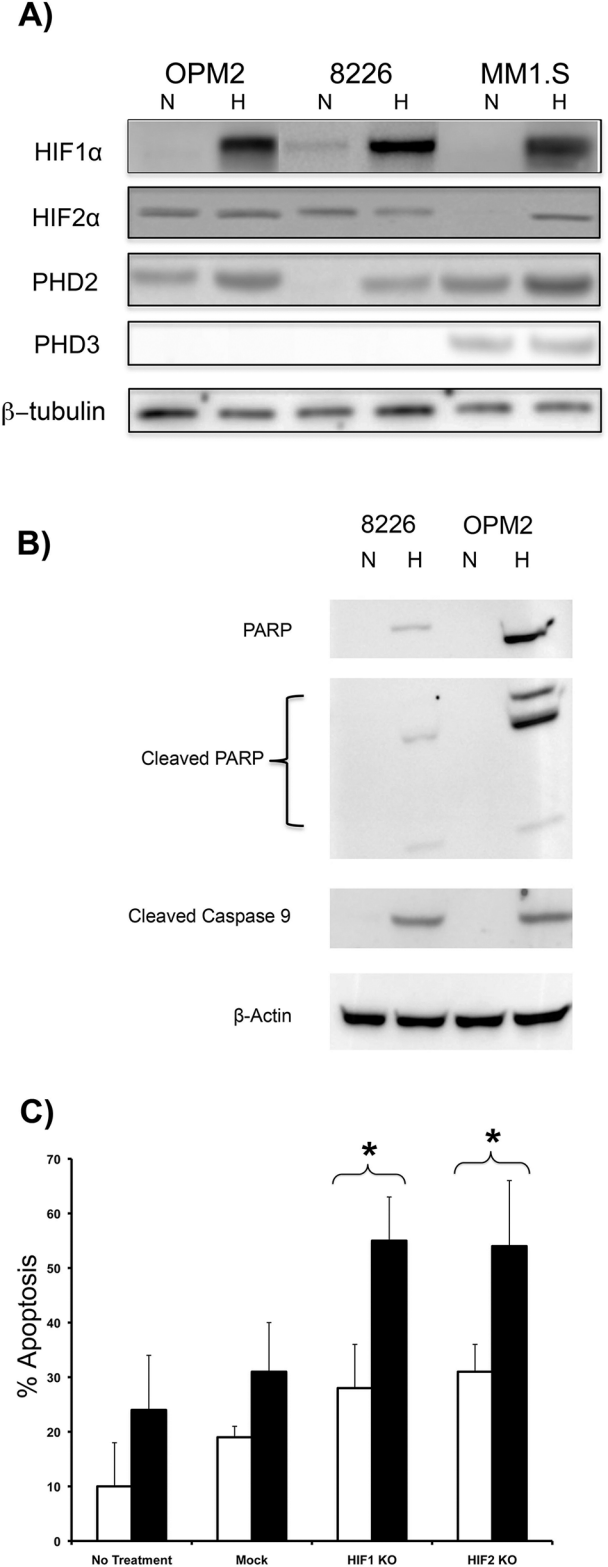
Characterization of O_2_-sensing pathway and HIFα-subunit expression in MM cell lines. (A) Immunoblots of HIF1α, HIF2α, PHD2 and PHD3 expression in OPM2, 8226 and MM1.S cell lines cultured under normoxic (22% O_2_) or hypoxic (0.1% 0_2_) conditions for 48 hrs. (B) Immunoblots of cleaved PARP and cleaved caspase 9 in 8226 and OPM2 cells. cultured under normoxic (22% O_2_) or hypoxic (0.1% 0_2_) conditions for 48 hrs. (C) Knockdown of HIFα-subunits sensitizes 8226 cells to hypoxia-mediated apoptosis. 8226 cells were transfected with HIF1α siRNA, HIF2α siRNA or non-specific scrambled control siRNA and then cultured under normoxic (22% O_2_ white bars) or hypoxic (0.1% 0_2_ black bars) conditions for 48 hrs and apoptosis was measured by flow cytometry for cleaved caspase-3. The data shows mean ± SEM of 3 independent experiments * P<0.05.

We previously analyzed the expression patterns for HIF1α- and HIF2α-subunits in a panel of MM cell lines and found that HIF1α expression was mostly O_2_-dependent and expression levels were strongly induced by hypoxia, whilst HIF2α tended to be constitutively expressed (under both normoxic and hypoxic conditions) and this tended to be independent of O_2_ levels [9] (Fig 1A). This implicated that differential regulation of the α-subunits is due to specific variations in the O_2_-sensing pathways so we assayed for expression of the two most common PHD isoforms, PHD2 and PHD3. We found that PHD2 was expressed at very low levels in 8226, but constitutively expressed in OPM2 and MM1.S cells under normoxic conditions. Since PHD2 preferentially hydroxylates and initiates degradation of HIF1α [21] the absence of PHD2 explains why 8226 cells express low, but constitutive levels of the HIF1α-subunit under normoxic conditions, although hypoxic conditions markedly increased PHD2 expression in these cells (Fig 1A). We also found that PHD3, which preferentially hydroxylates HIF2α [22], was absent in both 8226 and OPM2 cells thereby explaining the constitutive expression of HIF2α under normoxic conditions. In contrast, PHD2 and PHD3 were both expressed in MM1.S cells and as such, both of the HIFα-subunits are correspondingly sensitive to O_2_ (Fig 1A). We next asked if changes in HIFα expression under hypoxic conditions correlated to sensitivity to apoptosis in either 8226 or OPM2 cell lines. As shown in Fig 1B, OPM2 cells were markedly more sensitive than 8226 to apoptosis (measured by cleavage of PARP and caspase 9) that was induced by culturing the cells under hypoxic conditions, perhaps because both HIF1α and HIF2α are constitutively expressed in 8226 cells. We tested this by using siRNA to target either HIF1α or HIF2α in 8226 cells (as described in [9]) and assayed the impact of silencing each subunit on the regulation of sensitivity to hypoxia. We have previously shown that siRNA silenced their targeted HIF gene by approximately 75–90% under both normoxic and hypoxic conditions. As shown in Fig 1C, silencing either of the individual HIFα-subunits had little or no effect on apoptosis in 8226 cells cultured under normoxic conditions (22% O_2_, 48 hours), but significantly enhanced killing of 8226 cultured under low pO_2_ conditions (0.1%, 48 hours), supporting our hypothesis that downregulation of PHD3 may regulate sensitivity to hypoxia by the constitutive of induction of HIF2α.

### Restoration of PHD3 expression modulates HIF2α expression and activity

**Fig 2:**
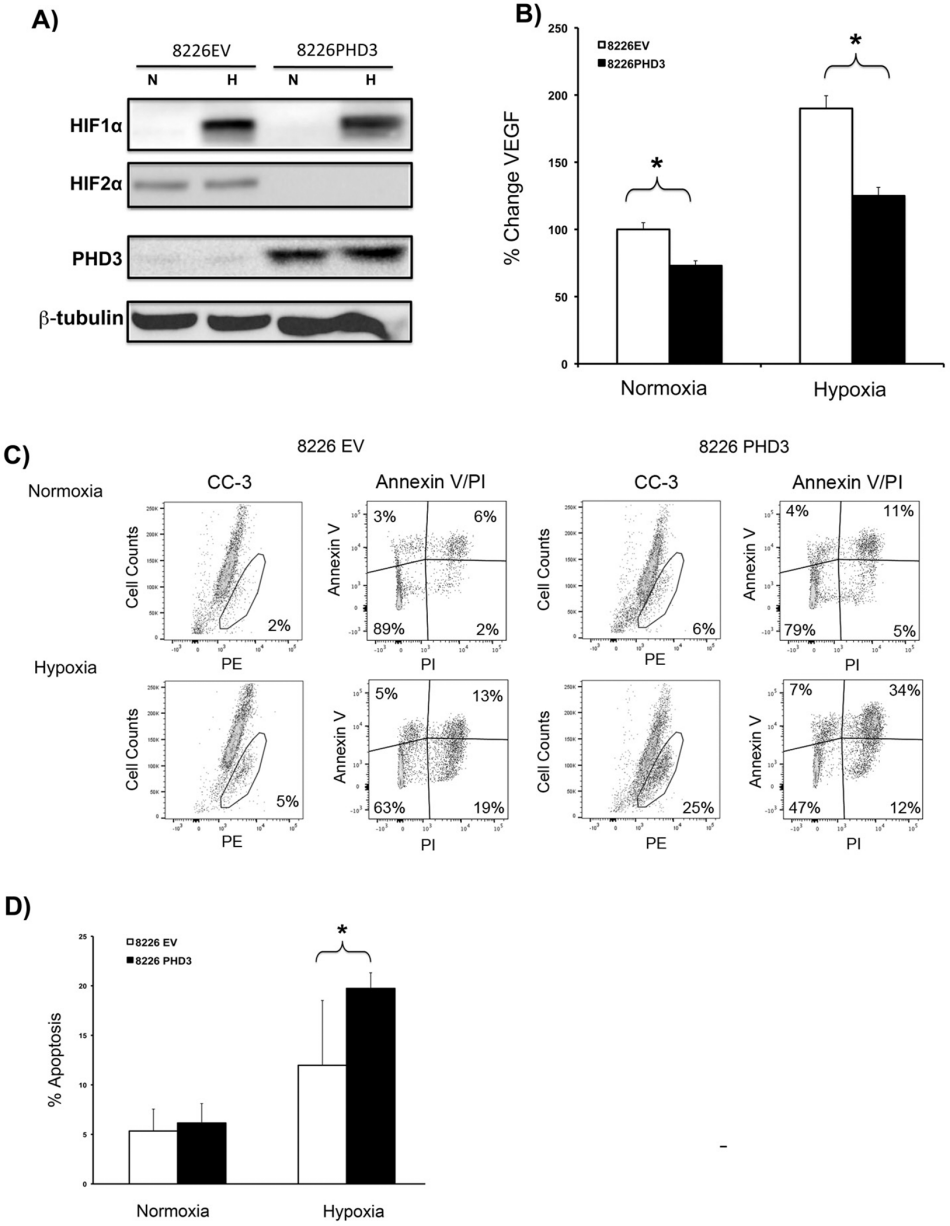
Exogenous expression of PHD3 expression inhibits HIF2α and restores sensitivity to hypoxia. (A) Immunoblots of HIF1α, HIF2α, and PHD3 expression in 8226 cells stably transduced with either empty vector (8226EV) or PHD3 (8226PHD3) lentivirus. Cells were cultured under normoxic (N) or hypoxic (H) conditions for 48hrs, and the cell lysate was collected and immunoblotted for indicated proteins. (B) PHD3 expression inhibits VEGF expression. Isogenic 8226 cells were cultured under normoxic or hypoxic conditions as described above and the supernatant was collected and VEGF levels measured by ELISA. Data is presented as % change in mean VEGF (pg/mg protein) ± STDEV of 3 independent experiments (bracket* = P<0.05). (C) Exogenous expression of PHD3 increases sensitivity of 8226 cells to hypoxia-mediated apoptosis. Representative histogram of isogenic 8226 cells cultured under normoxic conditions for 48 hrs. Apoptosis was measured by flow cytometry for cleaved caspase-3 kit. (D) Isogenic 8226 cells were cultured under normoxic or hypoxic conditions for 48 hrs and apoptosis was measured by flow cytometry for cleaved caspase-3. Values represent mean ± STDEV of % apoptosis of 3 independent experiments.

To further explore the role of PHD3 expression in MM cells, we generated isogenic 8226 using a *PHD3*-expressing lentivirus (8226PHD3) or empty vector control (8226EV) to test if restoring the PHD3 mediated O_2_-sensing pathway affected HIF2α expression and sensitivity to hypoxia-mediate apoptosis. Immunoblots presented in Fig 2A demonstrate that the stable transduction of 8226 cells with a PHD3-expressing lentiviral vector (8226PHD3) resulted in the specific downregulation of HIF2α (but not HIF1α) under both normoxic and hypoxic conditions. We believe that our strategy to exogenously express PHD3 was physiologically relevant since its restoration also impacted on the expression of vascular endothelial growth factor (VEGF), a known downstream hypoxia-induced target of HIF. As expected, culturing 8226EV cells under hypoxic conditions significantly (P<0.05) increased VEGF expression by 2 fold compared to cells cultured under normoxic conditions (Fig 2B white bars) but that hypoxia-mediated VEGF expression in 8226PHD3 cells was significantly decreased (P<0.05) (Fig 2B black bars). These data support the hypothesis that exogenous expression of PHD3 impacts not only on HIF2α regulation but also modifies the downstream HIF-mediated responses to hypoxia in these cell lines. Importantly, exogenous PHD3 expression also overcomes the resistance of 8226PHD3 cells to hypoxia-mediated apoptosis. As shown in Fig 2C (histogram of an individual flow cytometry experiment), 8226PHD3 cells were significantly sensitized to hypoxic-mediated apoptosis (an increase from ~10% killing to ~25% killing) compared to 8226EV measured by annexin V/PI (an early marker for apoptosis) and cleaved caspase-3 (a later marker for apoptosis) (Fig 2C). These results were confirmed in 3 additional independent experiments that are summarized in Fig 2D.

### Re-expression of *PHD3* mRNA with a DNA methyltransferase inhibitor sensitized MM cells to hypoxia

**Fig 3:**
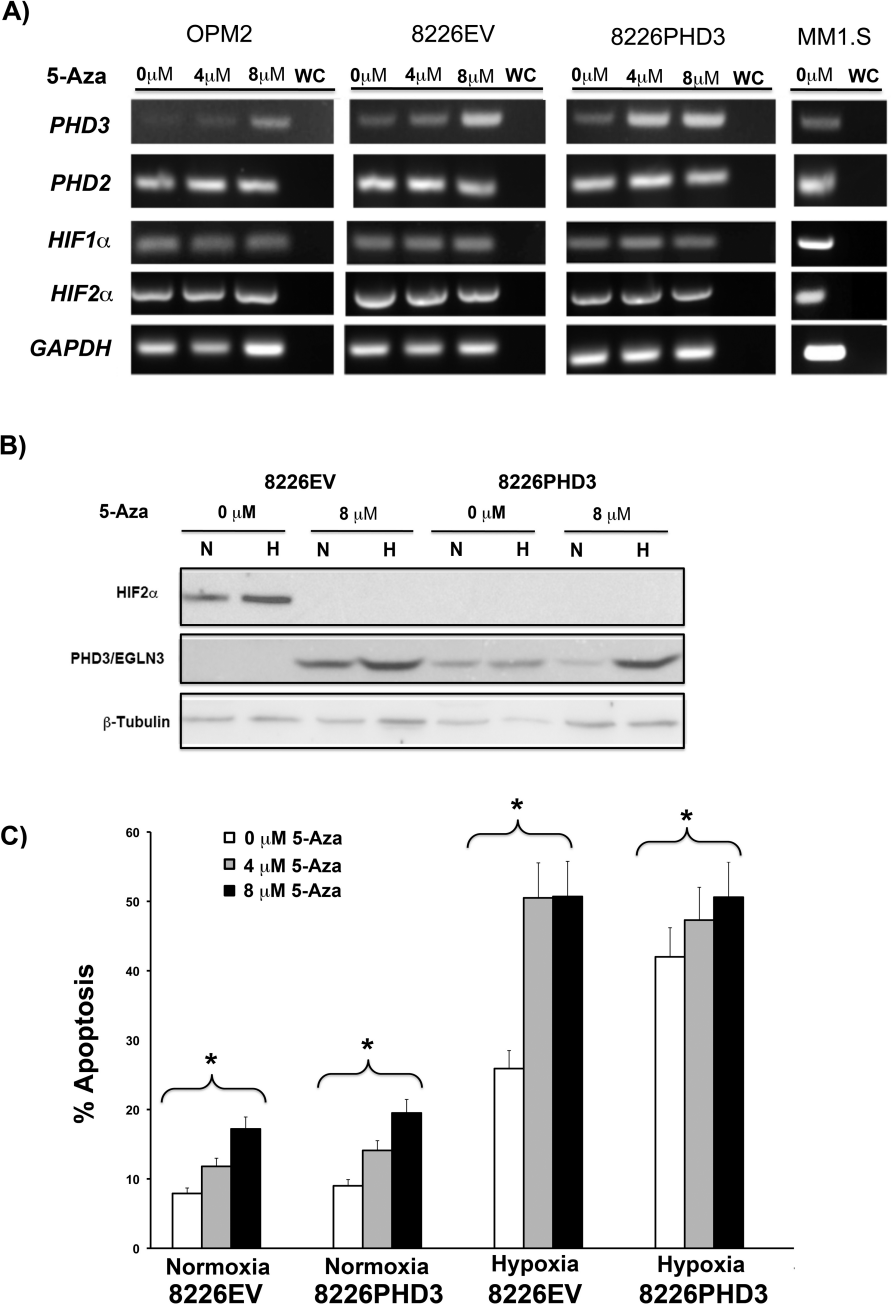
5-Aza-dC treatment reverses hypoxia resistance of MM cells. (A) 5-Aza-dC treatment restored expression of *PHD3* mRNA transcripts in various MM cell lines. (B) Treatment with 5-Aza-dC results in upregulation of PHD3 expression and concomitant downregulation of HIF2α protein. Immunoblot of isogenic 8226 cells treated with 5-Aza-dC under normoxic or hypoxic conditions for 48 hours. (C) 5-Aza-dC treatment sensitizes 8226EV cells to hypoxia-mediated apoptosis. Cells were cultured under normoxic (22% O_2_) or hypoxic (0.1% O_2_) conditions for 48 hrs in the presence or absence of indicated concentrations of 5-Aza-dC and apoptosis was measured by flow cytometry for cleaved caspase-3. The data shows mean ± SEM of 3 independent experiments. Brackets* = P<0.05.

Aberrant methylation of the *PHD3* promoter is observed in prostate, breast, melanoma and renal carcinoma cell lines [17]. In patient-derived plasma cell neoplasms, epigenetic silencing of *PHD3* (but not *PHD2*) also occurs frequently (~30% of cases) [19]. Thus, we asked if aberrant DNA methylation of the *PHD3* gene was responsible for its reduced expression in myeloma cell lines and if this could explain the O_2_-independence of HIF2α expression. We found that culturing the PHD3-negative cell lines, 8226 and OPM2, with the DNA methyltransferase inhibitor, 5-Aza-2′-deoxycytidine (5-Aza-dC), significantly increased the levels of *PHD3* mRNA expression compared to their respective untreated controls (Fig 3A). MM1S were used as a positive control for *PHD3* mRNA, as they are positive for PHD3 (Fig 1A). Furthermore, to control for non-specific effects of 5-Aza-dC on other oxygen-sensing factors, expression of *PHD2*, *HIF1*α and *HIF2*α mRNA were found to be unaffected by 5-Aza-dC treatments. In additional experiments 5-Aza-dC treatment of 8226 cells upregulated *PHD3* mRNA expression and protein expression with a concomitant downregulation of HIF2α, but not HIF1α (Fig 3B). Next we asked if restoring the PHD3 expression by 5-Aza-dC regulated the sensitivity of 8226EV cells to hypoxia-mediated apoptosis analogous to our experiments with PHD3 lentiviral transduced 8226PHD3 cells. As demonstrated in Fig 3C, culturing 5-Aza-dC treated 8226EV cells under hypoxic conditions (0.1%, 48hrs) significantly (P<0.05) induced apoptotic cell death, and this was comparable to the levels of hypoxia-mediated apoptosis observed in 8226PHD3 cells.

### Restoration of PHD3 expression does not impact sensitivity to bortezomib

**Fig 4:**
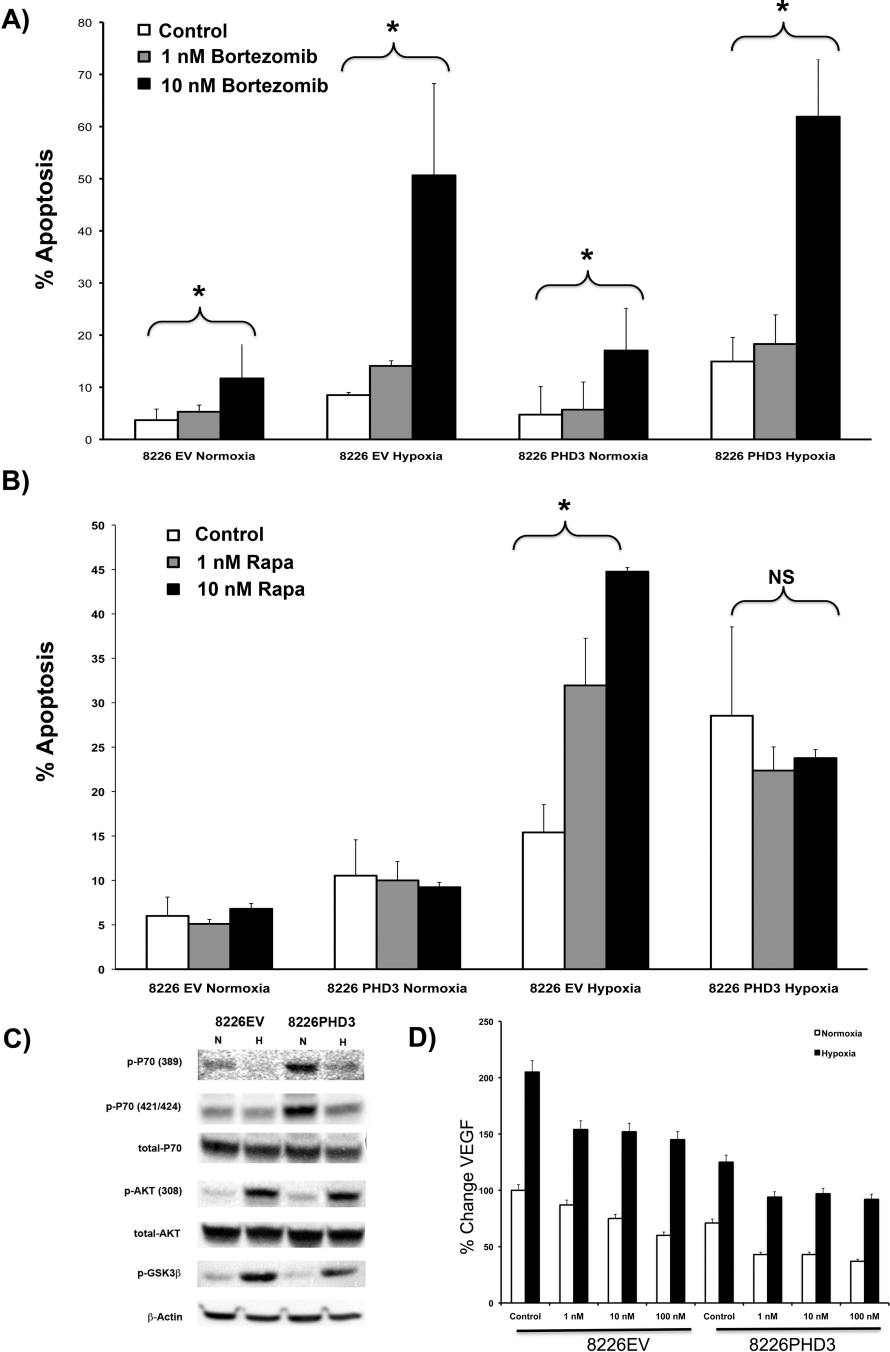
Effects of bortezomib or rapamycin treatment on the induction of apoptosis in myeloma cells cultured under hypoxic conditions. (A) Stably transduced 8226PHD3 or 8226EV control cells were cultured under normoxic (22% O_2_) or hypoxic (0.1% O_2_) conditions for 48 hrs in the presence or absence of indicated concentrations of bortezomib. Apoptosis was measured by flow cytometry for cleaved caspase-3. The mean ± SEM of 3 independent experiments is shown. Brackets* = P<0.05. (B) 8226PHD3 or 8226EV control cells were cultured under normoxic (22% O_2_) or hypoxic (0.1% O_2_) conditions for 48 hrs in the presence or absence of indicated concentrations of rapamycin as described above. Induction of apoptosis was measured by flow cytometry for cleaved caspase-3. The mean ± SEM of 3 independent experiments is shown. Brackets* = P<0.05. (C) Effects of hypoxia on expression of mTOR/AKT signaling pathway. 8226PHD3 or 8226EV control cells were cultured as described above and lysate collected and immunoblotted for indicated proteins. (D) Effects of hypoxia on VEGF expression. 8226PHD3 or 8226EV control cells were cultured under normoxic or hypoxic conditions as described above. VEGF levels were then measured in the supernatant by ELISA. Data is presented as % change in mean VEGF (pg/mg protein) ± STDEV of 3 independent experiments (bracket* = P<0.05).

It has been previously reported that in MM cells, low pO_2_ and activation of HIF confers a striking resistance to bortezomib-mediated killing that can be overcome by knocking down HIFα-expression with siRNA [7]. However, as shown in Fig 4A, we observed that hypoxic conditions had the opposite effect and significantly enhanced bortezomib-mediated apoptosis. In fact, these results were more in line with reports that hypoxia and bortezomib synergistically killed MM cells rather that protecting them from apoptosis [23, 24]. These are important results because if constitutive HIF expression protects MM cells from hypoxia-mediated resistance to bortezomib, we would expect that restoring the PHD3 O_2_-sensing pathways would overcome this resistance. However, we found that restoring PHD3 had no effect on bortezomib-mediated killing of MM cells under hypoxic conditions, suggesting that bortezomib and hypoxia may act independently from each other.

We previously demonstrated that targeting the mTOR signaling pathway with the mTOR inhibitor, rapamycin, in combination with a HIF/DNA targeting polyamide compound (HIF-PA), synergistically killed 8226 MM cells under both normoxic and hypoxic conditions [9, 25, 26]. We next asked if altering PHD3 expression regulated the sensitivity to rapamycin in our model. Surprisingly, culture of 8226PHD3 cells with rapamycin under low pO_2_ conditions did not significantly increase (P>0.05) the hypoxia-mediated apoptosis (Fig 4B), perhaps due to the fact that restoration of PHD3 (and concomitant degradation of HIF2α) alters the **transcription** of hypoxia-related genes, while rapamycin acts downstream by inhibiting of protein **translation**. As shown in Fig 4C, hypoxia induced similar levels of inhibition of P70, a downstream targets of mTOR, independent of PHD3 expression. Furthermore, culture of cells under hypoxic conditions, which is known to inhibit mTOR activity via REDD1 [27], increased phospho-AKT(308) phosphorylation and activation of GSK3β, a result which was previously observed in rapamycin-treated 8226 cells [28]. Finally, we found that VEGF expression was synergistically inhibited in 8226PHD3 cells cultured with rapamycin under hypoxic conditions. Altogether, the above data suggest that the differential response of 8336PHD3 cells to co-culture with hypoxia and rapamycin is not due to PHD3-mediated alterations in the mTOR-signaling pathway. This provides further evidence that restoration of PHD3 regulates sensitivity to hypoxia acts specifically through inhibition of HIF2α-mediated gene **transcription**.

A critical aspect of tumor oncogenesis is the need to maintain the physiological and biochemical requirements necessary for cell survival that are perturbed as a result of unregulated growth of cancer cells. In fact, there is increasing evidence that rather than simply being a physiologic stressor that tumor cells need to overcome or bypass, low pO_2_ conditions may actually be supportive of tumor growth, progression and the development of resistance to chemotherapy, and that this is dependent upon, at least in part, by HIF. A critical component of HIF is the concomitant requirement that cells are able to sense and respond to reduced O_2_ levels in order to activate the adaptive hypoxic response. Thus, while HIF’s ability to regulate the adaptive hypoxic response has been well studied in many cancer models, the roles played by the O_2_-sensing PHD isoforms is much less clear, despite evidence that they may be critical for both O_2_-dependent and O_2_-independent regulation of tumorgenesis.

We believe that PHD activity likely plays a greater role in cancer cells then simply hydroxylating and targeting HIFα-subunits for proteasome degradation. In fact, the heterogenous expression patterns of the various PHD isoforms and the preferential affinity for the different α-subunits suggests that PHD expression and activity may play a critical, yet poorly defined role in regulating the adaptive responses to hypoxia in various solid and hematological tumors. For example, over expression of PHD1 suppresses HIF-1α accumulation and colon cancer growth [29], whilst immortality of human endometrial cancer cells is often associated with loss of PHD2 [30]. Likewise, PHD3 has been shown to act as a pro-apoptosis factor [10], and downregulated PHD3 expression is frequently observed in glioblastoma [11, 12], colorectal cancer [31], soft tissue sarcomas [14] and in myeloma [19]. On the other hand, PHD3 expression was noted to be higher in well-differentiated pancreatic tumors and that metastatic lesions exhibited less PHD3 than the average cancer sample, indicating a gradual loss of PHD3 mRNA during the process of tumor de-differentiation and metastasis [16]. In our isogenic model, the re-expression of PHD3 restored both O_2_-dependent inhibition of HIF2α as well as the sensitivity of these cells to hypoxia when compared to isogenic empty vector control cells. We also found that rescuing PHD3 expression with the demethyltransferase inhibitor, 5-Aza-dC, inhibited HIF2α expression as well as also restoring sensitivity to hypoxia-mediated killing. In previous studies, Hatzimicheal et al ([19]) and Shah et al [18] observed frequent aberrant CpG methylation of *PHD3*, but not *PHD2*, in multiple myeloma (MM), Waldenstrom's macroglobulinaemia (WM) and monoclonal gammopathy of undetermined significance (MGUS*)*. Our data suggests that restoring PHD3 expression in MM cells could potentially be clinically efficacious. Importantly, 5-Aza-dC, has also been shown to have anti-myeloma activities [32, 33].

One important consideration about treating myeloma cells is that engraftment within the hypoxic bone marrow microenvironment (and the subsequent activation of HIF) may actually protect these cells from chemotherapy. In fact, it was recently reported that hypoxia conferred resistance to bortezomib-mediated apoptosis in MM cell lines and inhibiting HIF1α expression restored sensitivity [7]. However we found that culturing cells under hypoxic conditions not only did not protect against bortezomib-mediated apoptosis but that there was a marked sensitization to bortezomib in the presence of hypoxia (Fig 4A). Furthermore, this hypoxia-mediated sensitization occurred whether or not PHD3 was expressed in these cells. A possible explanation is that the anti-MM effects of bortezomib act on pathways that are independent of PHD3/HIF2α activity. These findings have important considerations for the clinic, as targeting HIF activity may not potentiate or overcome the anti-MM effects of bortezomib in resistance tumor phenotypes.

In our previous studies we also noted a strong correlation between the anti-angiogenic effects of mTOR inhibition and the induction of hypoxia-mediated apoptosis *in vivo* and *in vitro* [9, 20, 25, 26, 34]. In this study, we observed that the isogenic 8226EV cell line was sensitized to hypoxia-mediated apoptosis by treatment with rapamycin (Fig 4B), but targeting mTOR did not significantly enhance the killing of 8226PHD3 cells cultured under hypoxic conditions. We believe that these phenomena were due to a cascade-like series of events, in which hypoxia-mediated activation of HIF and subsequent **transcription** of pro-survival and pro-angiogenic factors was blocked by the rapamycin-mediated inhibition of mTOR-mediated **translation** in 8226EV cells, resulting in cell death. On the other hand, restoring PHD3 and inhibiting HIF2α-mediated gene transcriptional events that occur upstream of rapamycin’s targeting of mTOR-mediated protein translation, may reduce the effectiveness of targeting mTOR in these cells.

## Conclusions

In summary, HIF and related hypoxic response factors are frequently upregulated in MM, and this has been implicated in contributing to the development and prognosis of myeloma tumors [35]. In this study, we show that PHD3 likely acts as a tumor suppressor and mediates a critical role in O_2_-dependent regulation of the HIF2α and sensitivity to hypoxia in MM cells. Importantly, rescuing this O_2_-sensing pathway was sufficient to restore sensitivity to hypoxia. Thus, in this sense, understanding and targeting the PHD O_2_-sensing pathways may have a much more important role in the survival and progression of MM, other than simply targeting the α-subunits for proteasome degradation. We further believe that understanding these roles and mechanisms of the oxygen-sensing pathway could be clinically relevant as biomarkers as well as developing novel therapies against patient MM engrafted in the hypoxic bone marrow environment.

## Supporting information

S1 File Supporting information data file. (XLSX)
